# Supplementary material for: Bigger Is Fitter? Quantitative Genetic Decomposition of Selection Reveals an Adaptive Evolutionary Decline of Body Mass in a Wild Rodent Population
Source: PLoS Biol. 2017 Jan 26;15(1):e1002592. doi: 10.1371/journal.pbio.1002592 (PMC5268405; doi:10.1371/journal.pbio.1002592)
Supplement: S1 Table — Means and standard deviations are given for adults. Selection parameters were obtained from a multi variate animal model containing body mass in g, body and tail length in mm, as well as rLRS as a measure of fitness. Credibility intervals should be interpreted with care because fitness was modelled as a Gaussian trait in order to obtain meaningful selection parameters, while its distribution is closer to a Poisson distribution. (PDF) [file pbio.1002592.s001.pdf]

Supplementary information for Bigger Is Fitter? Quantitative Genetic Decomposition of Selection Reveals an Adaptive Evolutionary Decline of Body Mass in a Wild Rodent Population

Bonnet Timothée[1,*], Wandeler Peter[1,2], Camenisch Glauco[1], Postma Erik[1,3]

1 Department of Evolutionary Biology and Environmental Studies, University of Zurich, Zurich, Switzerland
2 Natural History Museum Fribourg, Fribourg, Switzerland
3 Centre for Ecology and Conservation, College of Life and Environmental Sciences, University of Exeter, Cornwall Campus, Penryn, United Kingdom

*** timothee.bonnet@ieu.uzh.ch**

Table S1 Selection differentials (measuring total selection) and gradients (measuring direct selection only) for body mass, body length and tail length, when considered in the same selection analysis.

Trait	Mean	Standard deviation	Selection differential	Selection gradient
Body mass	41.7 g	5.3 g	0.85 g<br>95%CI $[0.00; 1.95]$	0.079 $g^{-}1$<br>95%CI $[-0.084; 0.219]$
Body length	115.4 mm	4.5 g	1.23 mm<br>95%CI $[-0.04; 2.45]$	-0.026 $mm^{-}1$<br>95%CI$[-0.151; 0.097]$
Tail length	55.6 mm	5.8 g	0.85 mm<br>95%CI $[0.03; 1.85]$	0.037 $mm^{-}1$<br>95%CI $[-0.062; 0.131]$

Means and standard deviations are given for adults. Selection parameters were obtained from a multi-variate animal model containing body mass in grams (g), body length in millimeters (mm) and tail length in mm as well as relative lifetime reproductive success as a measure of fitness. Credibility intervals should be interpreted with care because fitness was modelled as a Gaussian trait, in order to obtain meaningful selection parameters, while its distribution is closer to a Poisson distribution.
