# Supplementary material for: Bigger Is Fitter? Quantitative Genetic Decomposition of Selection Reveals an Adaptive Evolutionary Decline of Body Mass in a Wild Rodent Population
Source: PLoS Biol. 2017 Jan 26;15(1):e1002592. doi: 10.1371/journal.pbio.1002592 (PMC5268405; doi:10.1371/journal.pbio.1002592)
Supplement: S2 Table — All values are on the logit scale. Estimates are means of posterior distributions, 95% CI are highest posterior density credibility intervals, and pMCMC are posterior bayesian p-values. Potential adult mass was estimated from a growth model, along with birth date. The parameter “Interaction” refers to the interaction of potential adult mass with the time interval between the onset of winter and estimated birth date. (PDF) [file pbio.1002592.s002.pdf]

Supplementary information for Bigger Is Fitter? Quantitative Genetic Decomposition of Selection Reveals an Adaptive Evolutionary Decline of Body Mass in a Wild Rodent Population

Bonnet Timothée[1,*], Wandeler Peter[1,2], Camenisch Glauco[1], Postma Erik[1,3]

1 Department of Evolutionary Biology and Environmental Studies, University of Zurich, Zurich, Switzerland
2 Natural History Museum Fribourg, Fribourg, Switzerland
3 Centre for Ecology and Conservation, College of Life and Environmental Sciences, University of Exeter, Cornwall Campus, Penryn, United Kingdom

*** timothee.bonnet@ieu.uzh.ch**

Table S2 Outcome of the logistic regression of juvenile overwinter survival on potential adult mass and time between birth and winter.

Parameters	Estimates	95% CI	p_{MCMC}
Intercept	12.41	2.48; 23.00	0.005
Sex (male)	−1.07	−1.45; −0.68	< 0.001
Potential adult mass	−0.30	−0.58; −0.06	0.007
Days winter-birth	−0.11	−0.20; −0.02	0.006
Interaction	0.0025	0.0001; 0.0048	0.008

All values are on the logit scale. Estimates are means of posterior distributions, 95% CI are highest posterior density credibility intervals and p_{MCMC} are posterior bayesian p-values. Potential adult mass was estimated from a growth model, along with birth date. The parameter Interaction refers to the interaction of potential adult mass with the time interval between the onset of winter and estimated birth date.
