# Supplementary figures and images for: Bigger Is Fitter? Quantitative Genetic Decomposition of Selection Reveals an Adaptive Evolutionary Decline of Body Mass in a Wild Rodent Population
Source: PLoS Biol. 2017 Jan 26;15(1):e1002592. doi: 10.1371/journal.pbio.1002592 (PMC5268405; doi:10.1371/journal.pbio.1002592)

**Number of individuals**

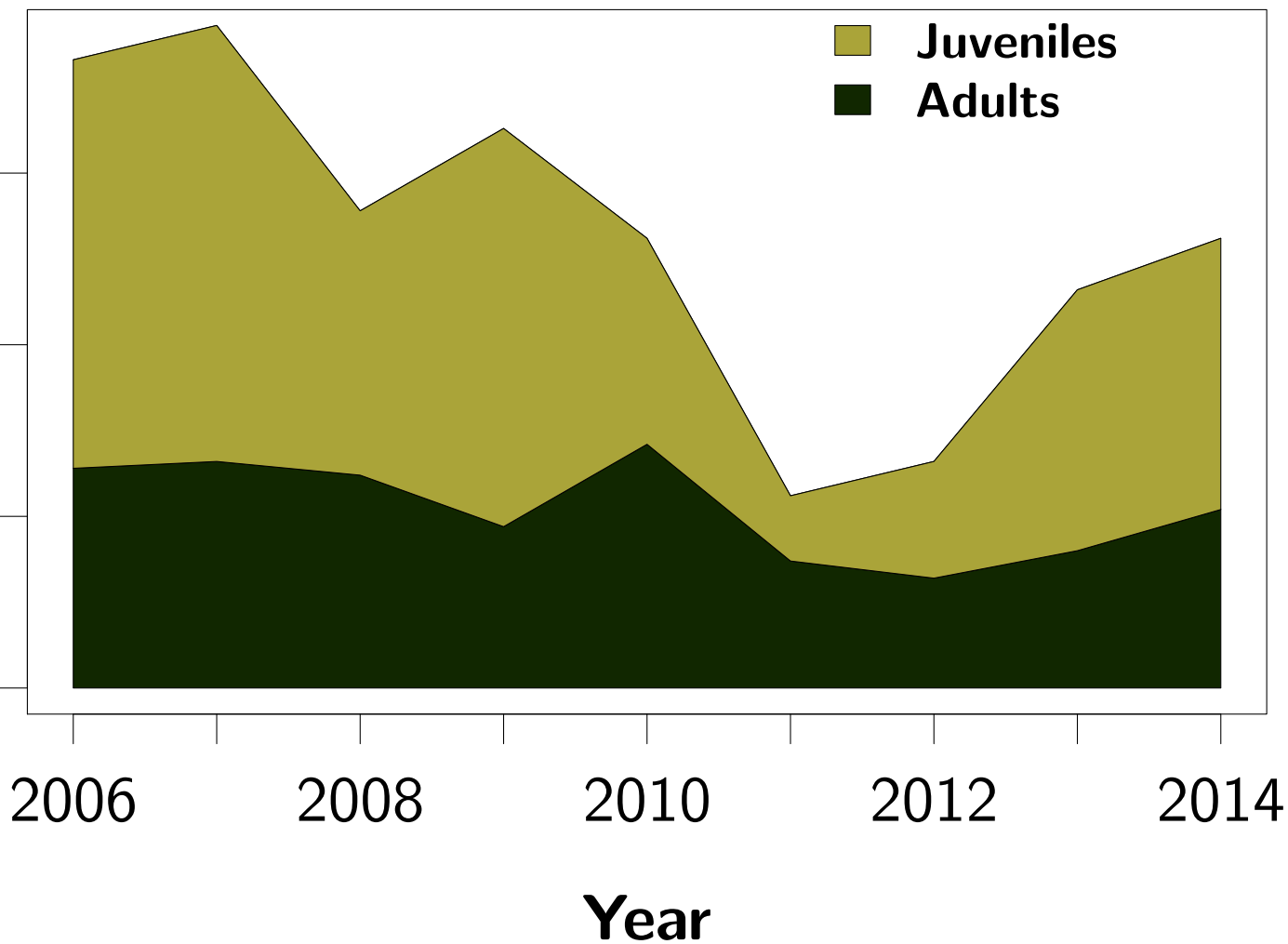

Supplement: S1 Fig — Number of unique adults and of juveniles captured in each y. (PDF) [file pbio.1002592.s003.pdf]

density of posterior probability

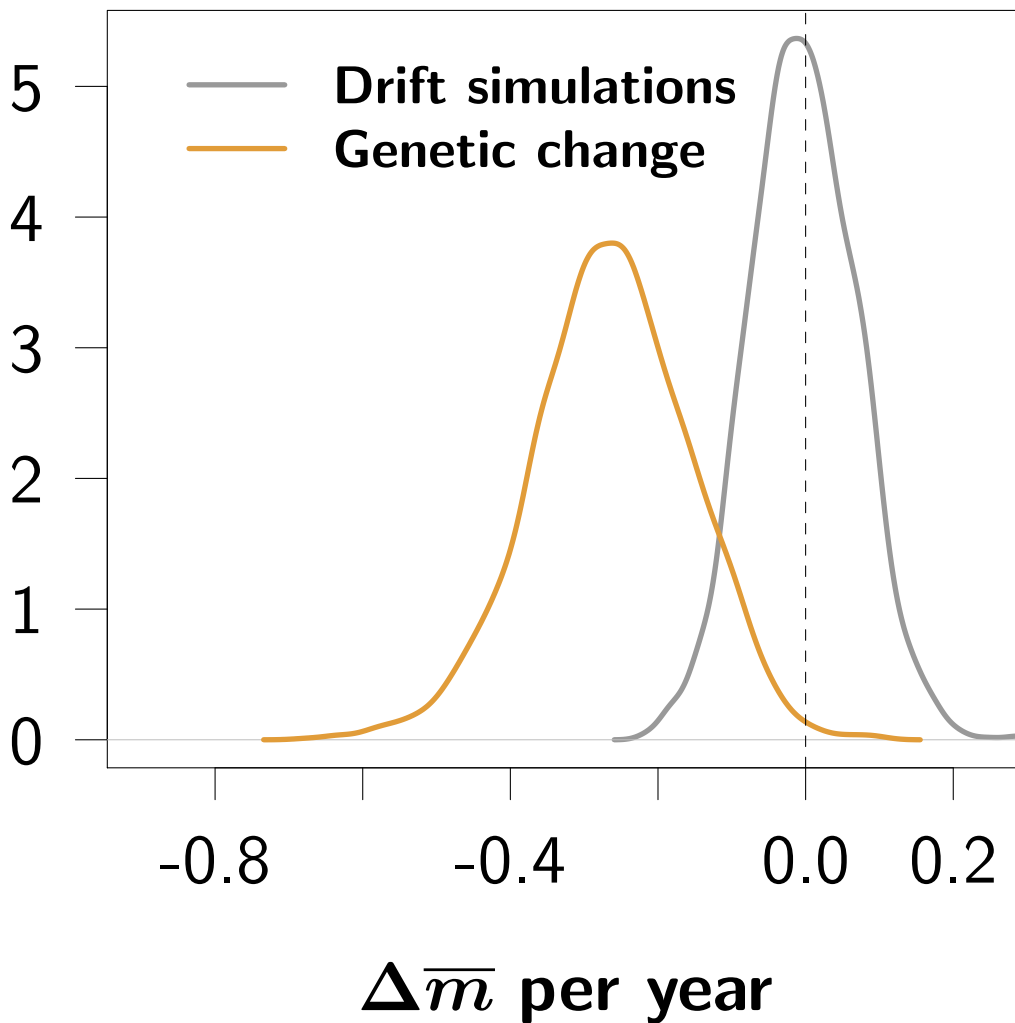

Supplement: S2 Fig — The posterior distributions of the realized rate of genetic change, estimated by the Price equation, exceeds that expected under genetic drift p = 0.009. Note that the posterior samples of drift and of genetic change are paired and correlated: small (/large) values of change due to drift are simulated for small (/large, respectively) posterior samples of estimated rate of evolution. Therfore, there is some overlap between the two distributions, but the probability that the genetic change was produced by drift is small (0.009). (PDF) [file pbio.1002592.s004.pdf]

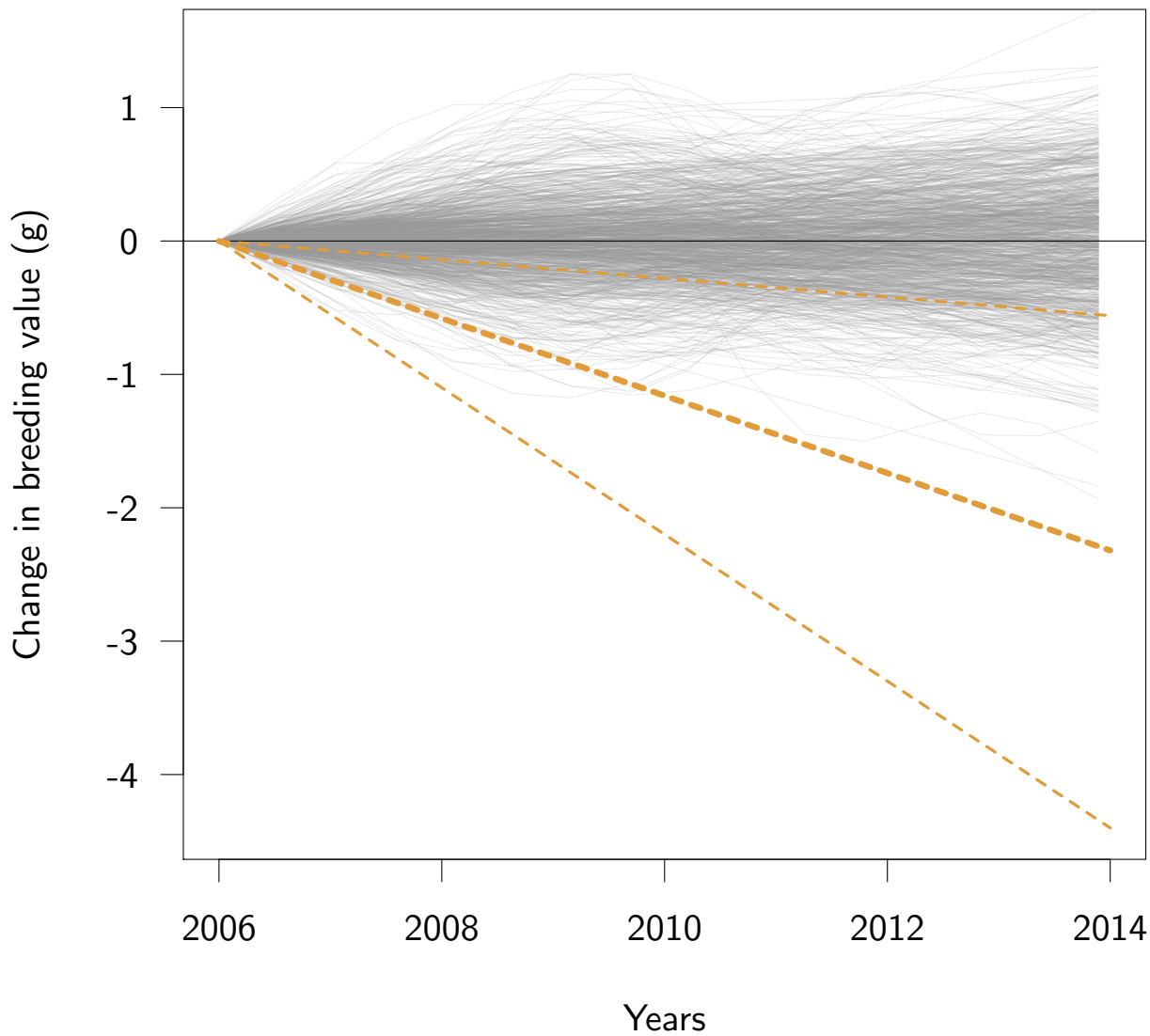

Supplement: S3 Fig — Evolution of breeding values for mass are shown relative to the year 2006. The yellow lines show the mode and 95% CI of the rate of evolution estimated by the Price equation within an animal model. The gray lines show 1,000 simulations of genetic drift, based on the real population pedigree and on the posterior distribution of genetic variance for mass estimated by the animal model. The probability that the observed rate of evolution happened because of drift is only 0.009, less than could be understood from the overlap between the two distributions. It is, however, important to notice that the two distributions are not independent, but that small (/large) values of change due to drift are simulated for small (/large, respectively) posterior samples of estimated rate of evolution. (PDF) [file pbio.1002592.s005.pdf]

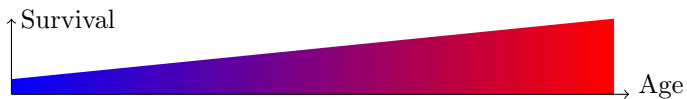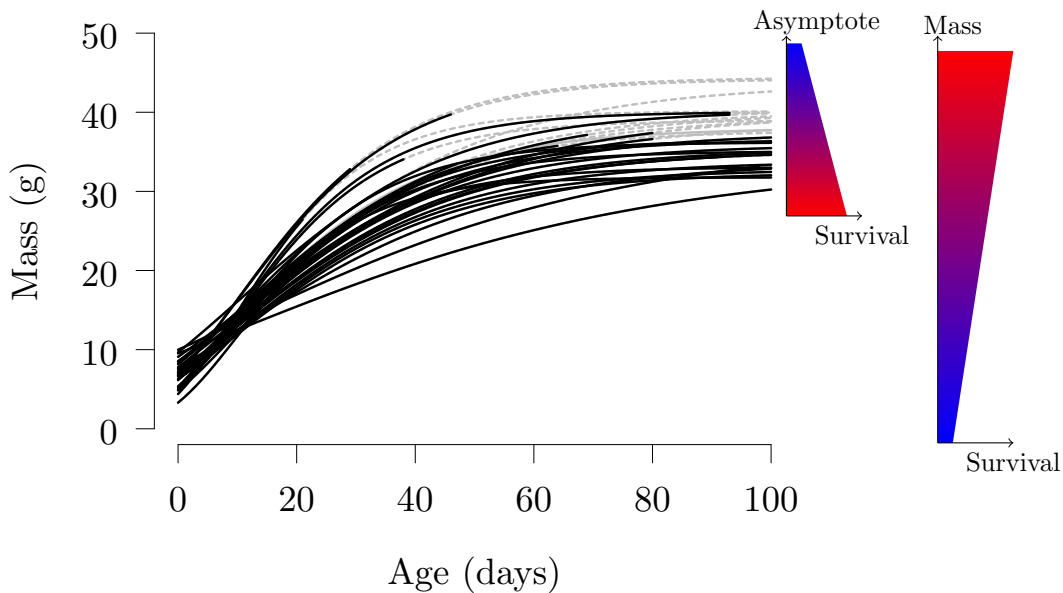

Supplement: S4 Fig — Black lines represent simulated individual growth trajectories from conception, and they are prolonged by gray dashed lines after an individual’s death. Color gradients indicate an increase in the parameter value from blue to red. The probability of surviving between the time of measurement and the next year increases with age. Because mass increases with age, there is apparent selection favoring heavier individuals. However, it is still possible for viability selection at a given developmental stage, such as asymptotic mass, to be negative. Because genetic variation is related to asymptotic mass, but not to age, the expected genetic change will be toward lower masses. (PDF) [file pbio.1002592.s006.pdf]

**(A)**

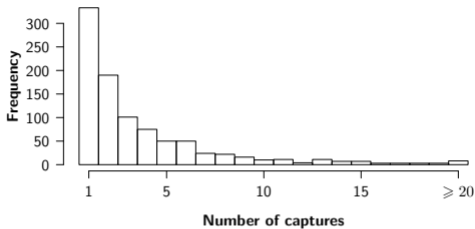

**(B)**

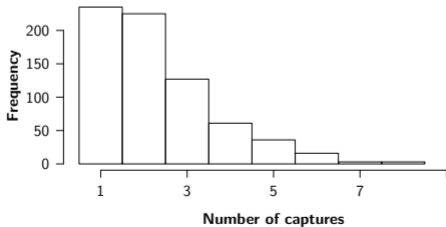

Supplement: S5 Fig — (A) for the whole data set; (B) for juveniles only. (PDF) [file pbio.1002592.s007.pdf]
