# Supplementary material for: Bigger Is Fitter? Quantitative Genetic Decomposition of Selection Reveals an Adaptive Evolutionary Decline of Body Mass in a Wild Rodent Population
Source: PLoS Biol. 2017 Jan 26;15(1):e1002592. doi: 10.1371/journal.pbio.1002592 (PMC5268405; doi:10.1371/journal.pbio.1002592)
Supplement: S1 Data — See the readme file for explanations on the content. (ZIP) [file pbio.1002592.s008.zip › DataAndCode/README.html]

# Data for the manuscript “Bigger Is Fitter? Quantitative Genetic Decomposition of Selection Reveals an Adaptive Evolutionary Decline of Body Mass in a Wild Rodent Population”

This repositery provides all the data necessary to reproduce the results in the manuscript.

1. AllM.txt contains all vole identity, sex, age, date, location, measurment…
2. ped.txt contains the pedigree of the population.
3. SnowData.txt contains the date of the first and last snow free day of each year. Numbers are julian days.
4. DistributionCaptures.txt contains the number of capture per individual.
5. DistributionJuvenilesCaptures.txt contains the number of capture per individual, during their first year of life.
6. popsize.txt contains the number of juveniles and adult voles per year.
7. PhenotypicMeanMass.txt contains for each year the estimate and confidence interval of the phenotypic mass corrected for age and sex.
